# Early and Strong Leptin Reduction Is Predictive for Long-Term Weight Loss during High-Protein, Low-Glycaemic Meal Replacement—A Subanalysis of the Randomised-Controlled ACOORH Trial

**DOI:** 10.3390/nu14122537

**Published:** 2022-06-18

**Authors:** Kerstin Kempf, Martin Röhling, Winfried Banzer, Klaus Michael Braumann, Martin Halle, Nina Schaller, David McCarthy, Hans Georg Predel, Isabelle Schenkenberger, Susanne Tan, Hermann Toplak, Stephan Martin, Aloys Berg, ACOORH Study Group

**Affiliations:** 1 West-German Centre of Diabetes and Health, Dusseldorf Catholic Hospital Group, 40591 Dusseldorf, Germany; martin.roehling@vkkd-kliniken.de (M.R.); stephan.martin@vkkd-kliniken.de (S.M.); 2 Department of Sports Medicine, Institute for Sports and Sport Science, University of Frankfurt, 60487 Frankfurt, Germany; banzer@med.uni-frankfurt.de; 3 Department of Sports and Movement Medicine, Faculty of Psychology and Human Movement Sciences, University of Hamburg, 20148 Hamburg, Germany; braumann@uni-hamburg.de; 4 Department of Prevention, Rehabilitation and Sports Medicine, Klinikum Rechts der Isar, Technical University of Munich (TUM), 80992 Munich, Germany; martin.halle@mri.tum.de (M.H.); nina.schaller@mri.tum.de (N.S.); 5 DZHK (German Centre for Cardiovascular Research), Partner Site Munich Heart Alliance, 80992 Munich, Germany; 6 Public Health Nutrition Research Group, London Metropolitan University, London N7 8DB, UK; hdavid.mccarthy@gmail.com; 7 Institute of Cardiovascular Research and Sports Medicine, German Sport University Cologne, 50933 Cologne, Germany; predel@dshs-koeln.de; 8 KARDIOS, Cardiologists in Berlin, 10787 Berlin, Germany; schenkenberger@klinische-forschung-berlin.de; 9 Department of Endocrinology, Diabetes and Metabolism, Division of Laboratory Research, University Hospital Essen, University Duisburg-Essen, 45122 Essen, Germany; susanne.tan@uk-essen.de; 10 Department of Medicine, Division of Endocrinology, Medical University of Graz, 8010 Graz, Austria; hermann.toplak@medunigraz.at; 11 Faculty of Medicine, Heinrich Heine University Dusseldorf, 40591 Dusseldorf, Germany; 12 Faculty of Medicine, University of Freiburg, 79117 Freiburg, Germany; berg.aloys@web.de

**Keywords:** leptin, protein-rich, low-glycaemic meal replacement, weight reduction, multicentre study, RCT

## Abstract

Lifestyle interventions including meal replacement are suitable for prevention and treatment of obesity and type-2-diabetes. Since leptin is involved in weight regulation, we hypothesised that a meal replacement-based lifestyle intervention would reduce leptin levels more effectively than lifestyle intervention alone. In the international, multicentre, randomised-controlled ACOORH-trial (Almased-Concept-against-Overweight-and-Obesity-and-Related- Health-Risk), overweight or obese participants with metabolic syndrome criteria (*n* = 463) were randomised into two groups and received telemonitoring devices and nutritional advice. The intervention group additionally used a protein-rich, low-glycaemic meal replacement. Data were collected at baseline, after 1, 3, 6, and 12 months. All datasets providing leptin data (*n* = 427) were included in this predefined subanalysis. Serum leptin levels significantly correlated with sex, body mass index, weight, and fat mass at baseline (*p* < 0.0001). Stronger leptin reduction has been observed in the intervention compared to the control group with the lowest levels after 1 month of intervention (estimated treatment difference −3.4 µg/L [1.4; 5.4] for females; −2.2 µg/L [1.2; 3.3] for males; *p* < 0.001 each) and was predictive for stronger reduction of body weight and fat mass (*p* < 0.001 each) over 12 months. Strongest weight loss was observed after 6 months (−5.9 ± 5.1 kg in females of the intervention group vs. −2.9 ± 4.9 kg in the control group (*p* < 0.0001); −6.8 ± 5.3 kg vs. −4.1 ± 4.4 kg (*p* = 0.003) in males) and in those participants with combined leptin and insulin decrease. A meal replacement-based lifestyle intervention effectively reduces leptin which is predictive for long-term weight loss.

## 1. Introduction

Leptin, a key-regulatory hormone, is predominantly released from cells in the adipose tissue to display saturation state in the brain [1]. Serum leptin levels are generally higher in females compared to males [2]. In a normal-weight state, low leptin levels are a starvation signal and elevated levels reduce food intake by suppressing appetite [3,4,5]. Vice versa, in the obese state serum leptin levels have been shown to be permanently elevated. This is defined as peripheral hyperleptinemia and causes leptin resistance in the hypothalamus [6]. Moreover, there is a strong interaction in the regulatory processes of leptin and insulin release, which also plays an important role in the regulation of body weight and metabolic control [7].

Lifestyle interventions are effective not only in the prevention [8,9,10] but also in the treatment of type-2-diabetes and up to diabetes remission [11,12,13]. Nevertheless, they have been criticized since the induced weight loss is often transient and generally followed by weight regain. This might be caused by leptin resistance, which inhibits appropriate appetite control [14]. The international, multicentre ALMASED-Concept- against-Overweight-and-Obesity-and-Related-Health-Risk (ACOORH) study [15,16,17,18,19] is a randomised-controlled trial designed to compare the effects of a meal replacement-based lifestyle intervention vs. lifestyle intervention alone in overweight or obese adults with risk factors of the metabolic syndrome. With an estimated treatment difference (ETD) of −3.2 kg [−4.0; −2.5] (*p* < 0.001), previously published data demonstrated significantly higher weight reduction in the meal replacement intervention group compared to the control group after 3 months [15] accompanied by significantly stronger reductions of insulin and inflammation markers [18]. Reduction of insulin levels and weight loss were highly correlated and strongest weight loss was observed in those participants with the most pronounced insulin decrease [18].

So far, it is unknown how protein-rich, low glycaemic meal replacements affect leptin levels in overweight or obese females and males with risk factors of the metabolic syndrome. Therefore, in this subanalysis of the ACOORH trial, we investigated the effect of a meal replacement intervention on the change in serum leptin and analysed the associations between changes in leptin and reductions of body weight and fat mass with respect to sex.

## 2. Materials and Methods

### 2.1. Study Design and Population

The ACOORH study is an international, multicentre randomised-controlled trial analysing the effect of a meal-replacement based lifestyle intervention in overweight or obese persons with components of the metabolic syndrome. Details had been published before [15,16,17,18,19]. In brief, individuals aged 21–65 years, with a body mass index (BMI) of 27–35 kg/m^2^ and/or a waist circumference of ≥88 (females) or ≥102 cm (males) and fulfilling at least one of the following criteria of the metabolic syndrome: (a) fasting blood glucose (FBG) 100–125 mg/dL, (b) triglycerides 150–400 mg/dL, (c) high-density lipoprotein (HDL)-cholesterol < 40 mg/dL, or (d) untreated systolic blood pressure of 140–160 mmHg or diastolic blood pressure of 90–100 mmHg or anti-hypertensive medication were eligible for participation. Participation was not possible if at least one of the following exclusion criteria was fulfilled: (i) diabetes mellitus with FBG ≥ 126 mg/dL or HbA1c ≥ 6.5% (≥48 mmol/mol) or diabetes-related medical history (e.g., antidiabetic drugs or medical records); (ii) total body weight >141 kg; (iii) acute infections; (iv) chronic diseases such as cancer, chronic obstructive pulmonary disease, asthma, chronic gut diseases, nephropathy, and kidney insufficiency with glomerular filtration rate <30 mL/min/1.73 m^2^, liver cirrhosis, psychoses, dementia, (v) plans to move to areas unserved by ACOORH; (vi) (planned) smoking cessation during the study phase; (vii) medication for active weight reduction; (viii) pregnancy or breast-feeding; and (ix) known intolerance with components of the used meal replacement.

The study was conducted in accordance with the Declaration of Helsinki, and was approved by the responsible ethics committees of all participating centres. The trial was registered at drks.de (no. DRKS00006811). With a 1:2 allocation ratio, participants were randomised into either a lifestyle intervention control group (*n* = 134) or a meal replacement-based lifestyle intervention group (*n* = 293). In January 2015, the first participant was included and the last examination was performed in August 2017. Out of the 463 participants of the initial ACOORH cohort, only those with baseline and at least one follow-up leptin value (*n* = 427) were considered in the present subanalysis.

### 2.2. Intervention and Meal Replacement Regimen

At baseline, as well as after 1, 3, 6, and 12 months, participants of both groups visited the study centre to receive nutritional counselling and guidance on increasing physical activity [15]. Both groups were equipped with pedometers and telemetric scales automatically transferring the data into a personalised online portal. The data collected (i.e., body weight, diet protocols, steps) were discussed during study visits and participants were motivated to achieve their individual goals (i.e., weight reduction, healthy lifestyle changes).

The study consists of 6 months of an intensive lifestyle intervention followed by a moderate intensive follow-up phase until month 12 as described previously [15]. The intervention group additionally received a high-protein, low glycaemic meal replacement (Almased; Almased-Wellness-GmbH, Oberding, Germany) and was instructed to replace all three main meals in the first week. In weeks 2–4, breakfast and dinner should be replaced, and finally, only dinner should be replaced until week 26. Information about the preparation of the meal replacement as well as general information about low-carbohydrate meals and their influence on blood glucose and insulin levels, hunger and weight loss were provided in an accompanying manual.

### 2.3. Outcomes and Measurements

During all study visits, anthropometrical data (BMI, body weight, fat mass, lean body mass) were measured and blood was collected as described before [15,16,17,18,19]. Leptin, fasting insulin, interleukin (IL)-6, and C-reactive protein (CRP) were analysed in an accredited medical laboratory (Synlab, Leinfelden, Germany) according to previous reports [20,21] and ratios for leptin/body weight, leptin/fat mass, insulin/body weight, and insulin/fat mass were calculated. Prediabetes was defined as previously described [16]. The assessors were blinded for group allocation. Adverse and serious adverse events were continuously reviewed as described [15] by an external monitor.

### 2.4. Statistics

Sample size calculation and its assumptions have been described before [15]. If not otherwise stated, intention-to-treat (ITT) analyses were performed. For imputation of missing values, the ‘last-observation-carried-forward’ (LOCF) principle was used. The present predefined subanalysis focuses on the tertiary outcome of sex-stratified changes of leptin compared between the control and the intervention group and their association with prediabetes, age, BMI, body weight, fat mass, lean body mass, fasting insulin, IL-6, and CRP. In order to analyse the influence of leptin levels on body weight changes, uniform tertile stratification was performed for the observed leptin reductions after 1 month (1st tertile = highest leptin reduction; 2nd tertile = medium leptin reduction; 3rd tertile = lowest leptin reduction) and was related to the weight reduction at all time points. Subsequently, tertile stratification was also applied to the achieved insulin reductions after 6 months (1st tertile = insulin reduction of >2 µU/mL; 2nd tertile = constant insulin values with changes ≤2 µU/mL; 3rd tertile = insulin increase >2 µU/mL) as described before [18] and the combined leptin and insulin tertiles were related to the weight reduction after 6 months. Non-parametric data were analysed with Mann-Whitney U, Wilcoxon, Kruskal-Wallis test with Dunn’s multiple comparison test, or Spearman correlation. Parametric data were evaluated with Student’s t-test, paired t-test, analysis of variance with repeated measures, or Person correlation. Multivariable linear regression analyses were performed to examine the associations between leptin or changes in leptin with body weight or weight changes with adjustment for potential confounders. All statistical tests were two-sided, and the level of significance was set at *p* = 0.05. The basic statistical analysis was performed by an independent institute (ACOMED Statistik^®^, Leipzig, Germany) not involved in the study execution. All analyses were performed using SPSS 22.0 (SPSS Inc., Chicago, IL, USA) and GraphPad Prism 6.04 (GraphPad Software, San Diego, CA, USA).

## 3. Results

### 3.1. Stronger Improvement of Leptin Levels, Body Weight and Fat Mass in the Intervention Group

**Figure 1:**
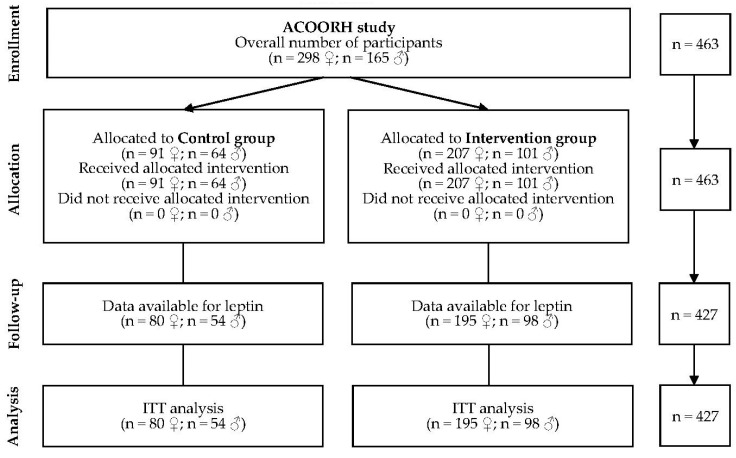
Flow chart. ITT: intention-to-treat.

Leptin data were available from 80 female and 54 male participants of the control group as well as from 195 female and 98 male participants of the intervention group and these data were used for ITT-analysis (Figure 1).

Baseline characteristics (Table 1) did not differ significantly between the control and intervention group. In participants with prediabetes serum leptin levels tended to be higher (19.5 µg/L [13.5; 27.7] vs. 16.7 µg/L [12.0; 24.3] in female and 7.8 µL/L [4.6; 9.9] vs. 6.5 µg/L [3.9; 9.7] in male participants).

**Figure 2:**
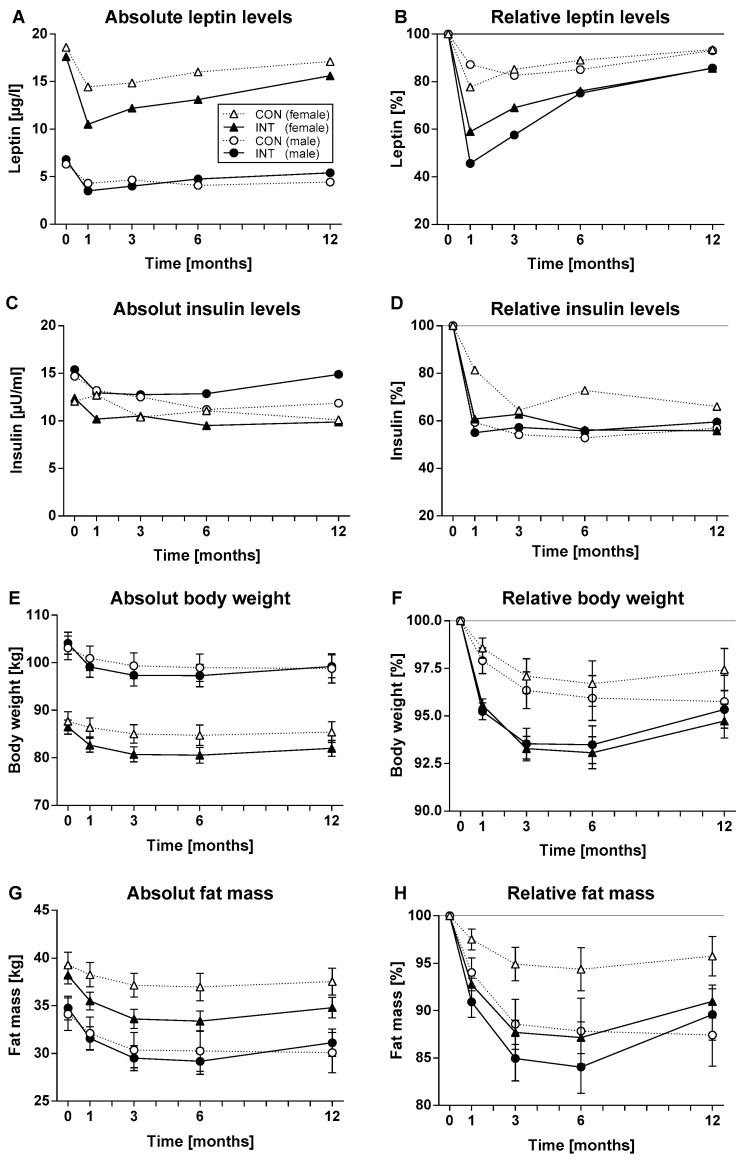
Shown are the courses of (**A**) absolute and (**B**) relative leptin levels in the control (CON) and the intervention (INT) group during the 6-month intervention and 6-month follow up phase separated for female (Δ CON, *n* = 80; ▲ INT, *n* = 195) and male (○ CON, *n* = 54; ● INT, *n* = 98) participants. (**C**) shows the course of absolute and (**D**) relative insulin levels, (**E**) absolute and (**F**) relative body weight, (**G**) absolute and (**H**) relative fat mass. Leptin and insulin data are shown as median, while body weight and fat mass data are shown as mean [95% confidence interval].

During the first month of the intervention, absolute leptin levels significantly decreased in all groups and started to re-increase during the subsequent study course (Figure 2A). At the final study visit, leptin levels were still significantly lower compared to baseline in females of the intervention group (*p* < 0.007). For both sexes, leptin reduction was rather transient and mostly pronounced after 1 month but was significantly more pronounced in the intervention group compared to the control group (ETD 3.4 µg/L [1.4; 5.4] for females; 2.2 µg/L [1.2; 3.3] for males; *p* < 0.001 each). Since the leptin levels were not normally distributed and leptin courses generally higher in females than in males, we normalized by calculating relative leptin levels in percent with baseline levels set to 100% (Figure 2B). This indicated that the courses of relative leptin levels were highly comparable between sexes. Relative leptin values also were lowest after 1 month and significantly lower in the intervention compared to the control groups (*p* < 0.0001 for both sexes).

Contrary to leptin, insulin courses were lower in females compared to males. Absolute and relative insulin levels (Figure 2C,D) became decreased in all groups and remained continuously lower compared to baseline (*p* < 0.0001 for both sexes in the intervention group and *p* < 0.01 in the control group).

Although females generally exhibited lower body weight than males, their fat mass was generally higher. Significant reductions of absolute body weight and fat mass were observed in all groups (within group comparisons: *p* < 0.0001 at all time points vs. baseline) with lowest values after 6 months, which corresponds to the end of the intense intervention phase. Until month 6, the reductions of body weight and fat mass (Figure 2E–H) were significantly stronger in the intervention than in the control group (*p* < 0.0001 for females, *p* < 0.01 for males; *p* < 0.0001 for females, *p* < 0.05 for males). Comparable observations were made for BMI (data not shown).

Absolute and relative values for leptin, insulin, body weight, and fat mass are shown in Figure 2.

### 3.2. Baseline Leptin Values Correlate with Baseline Parameters

Baseline leptin levels were positively associated with sex, prediabetic state, BMI, body weight, fat mass, lean body mass, and C-reactive protein. Sex-stratified analysis demonstrated that only BMI, body weight, fat mass, and fasting insulin levels were significantly correlated with baseline leptin levels in both sexes (Table 2).

### 3.3. Leptin Reduction Is Predictive for Loss of Body Weight and Fat Mass

Baseline leptin levels were significantly associated with baseline body weight and fat mass but not with their changes during the study. Vice versa, a strong predictive value could be proven for Δ leptin after 1 month and loss of weight and fat mass at all time points in both the control and the intervention group (Table 3).

### 3.4. The Leptin Decrease Is Only Partially Explained by Body Weight and Fat Loss and Stronger in the Intervention Group

**Figure 3:**
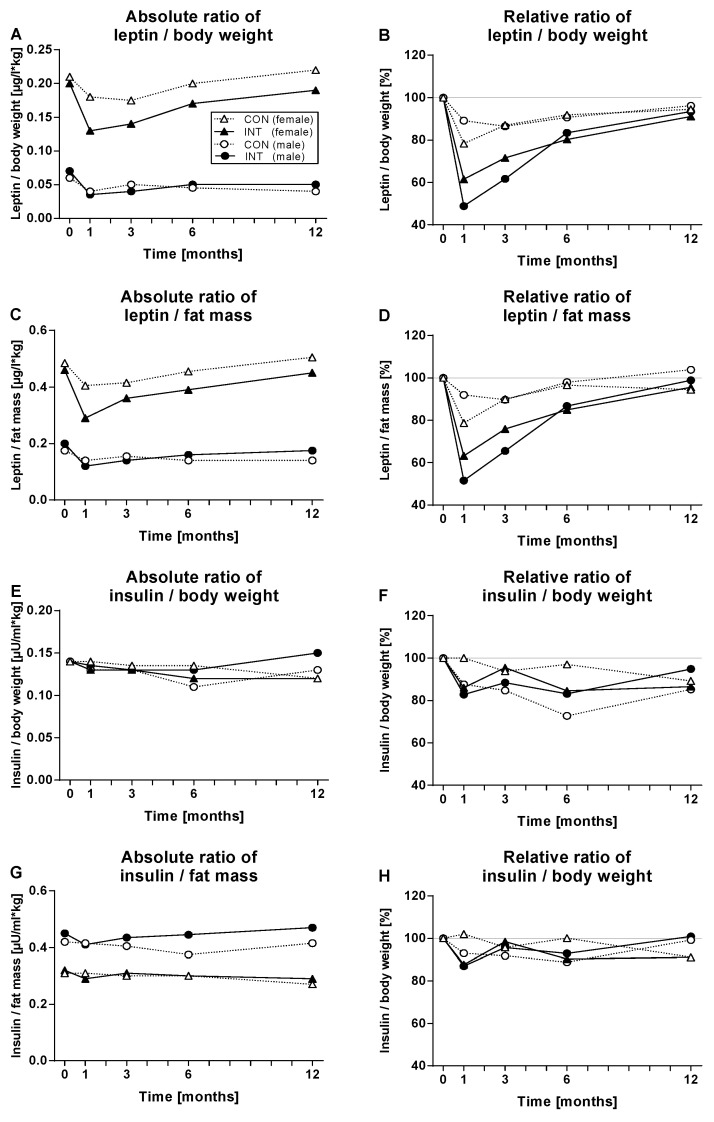
Shown are the median of (**A**) absolute and (**B**) relative ratios of leptin/body weight in the control (CON) and the intervention (INT) group during the 6-month intervention and 6-month follow up phase separated for female (Δ CON, *n* = 80; ▲ INT, *n* = 195) and male (○ CON, *n* = 54; ● INT, *n* = 98) participants. (**C**) shows the absolute and (**D**) relative ratios of leptin/fat mass, (**E**) absolute and (**F**) relative ratios of insulin/body weight, (**G**) absolute and (**H**) relative ratios of insulin/fat mass.

To find out if the reduction of leptin levels was related to the loss of weight and especially fat mass, the ratio of leptin to body weight (Figure 3) or to fat mass was calculated. Significant reductions of absolute ratios were observed until month six (*p* < 0.0001 for females, *p* < 0.01 for males in the intervention group, each).

Absolute and relative ratios for leptin/body weight, leptin/fat mass, insulin/body weight, and insulin/fat mass are shown in Figure 3. 

Regarding the relative ratios, after 1 month of the intervention the reduction of leptin was much stronger in relation to body weight or fatty mass loss, the effects were transient, and more pronounced in the intervention group (*p* < 0.0001 for females, *p* < 0.01 for males, each). However, the same analyses for insulin demonstrated that the observed reduction of insulin levels was nearly in parallel with the reductions of body weight and fat mass.

### 3.5. Leptin Reduction Accounts for Long-Term Weight Loss

**Figure 4:**
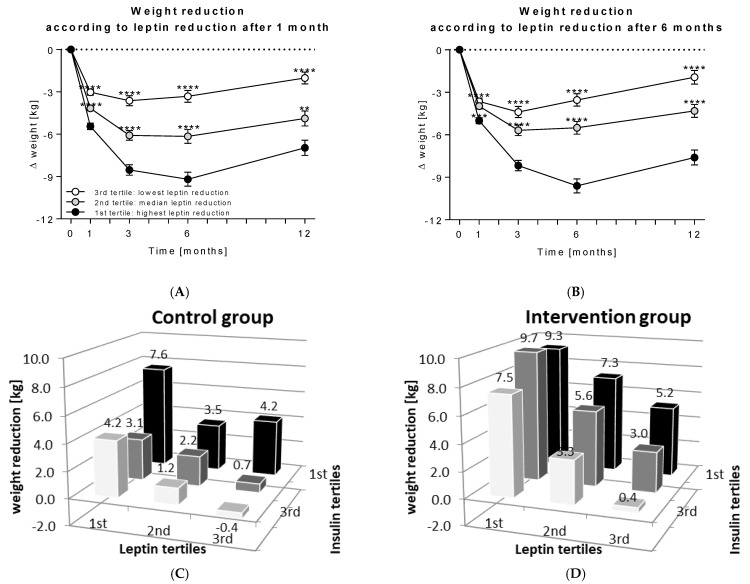
Participants of the intervention group (*n* = 293) were divided into three tertile groups according to their relative reduction (**A**) of leptin after 1 month (1st tertile = highest leptin reduction (*n* = 98); 2nd tertile = median leptin reduction (*n* = 97); 3rd tertile = lowest leptin reduction (*n* = 98)) and (**B**) of leptin after 6 months. Weight reduction was compared between tertile groups using Mann Whitney test with the 1st tertile as reference for each (**, *p* < 0.01; ***, *p* < 0.001; ****, *p* < 0.0001). The three leptin tertile groups were additionally stratified according to their insulin reduction after 6 months (1st tertile = insulin reduction of >2 µU/mL; 2nd tertile = constant insulin values with changes ≤2 µU/mL; 3rd tertile = insulin increase > 2 µU/mL) and weight reduction is shown (**C**) the control and (**D**) the intervention group.

The tertile analyses showed that those participants of the intervention group with the highest leptin reduction after 1 month also achieved the highest weight reduction during the study, while the group with the lowest leptin reduction already started to regain weight after 3 months (Figure 4A). The same was observed for participants with the highest leptin reduction after 6 months: The participants who maintained leptin reduction also maintained weight loss (Figure 4B).

Early leptin reduction accounts for long-term weight loss.

Combined tertile analyses, i.e., by dividing participants of the control or the intervention group, respectively, according to their relative leptin levels and then stratifying them by insulin change, demonstrated a relationship in leptin and insulin change on weight loss. In both the control and the intervention group, weight loss was generally higher in the 1st leptin tertile, as well as in the 1st insulin tertile. Participants with the strongest leptin and insulin reduction achieved mean weight reductions of 7.6 ± 4.4 kg (control group; Figure 4C) and 9.3 ± 5.0 kg (intervention group; Figure 4D).

## 4. Discussion

The international, multicentre, randomised-controlled ACOORH trial demonstrated that a high-protein, low glycaemic meal replacement-based lifestyle intervention is more effective on weight reduction than a control lifestyle intervention alone. Higher weight loss was accompanied by stronger reduction in leptin levels. Leptin reduction was transient with the lowest levels after 1 month of intervention. Although serum leptin levels were generally higher in females than in males, calculation of relative levels revealed comparative effects on leptin reduction for both sexes but generally stronger improvements in leptin, body weight, fat mass, and corresponding ratios in the intervention group. Baseline leptin levels were positively correlated with baseline body weight, BMI, fat mass, and fasting insulin in both sexes, while the leptin reduction within the first month was predictive for weight and fat mass loss over 12 months. Tertile analyses demonstrated that weight loss was most pronounced in those persons with the highest reduction of leptin and insulin.

Leptin is a protein hormone, predominantly secreted by cells from the adipose tissue, that regulates food intake and energy expenditure [1,3,4,5]. As demonstrated by our and previous studies [2,20,22,23], its plasma concentrations are proportional to fat mass and—maybe due to their higher amount of fat mass—generally higher in females compared to males [2]. Circulating leptin enters the brain through the blood–brain barrier and causes anorexia by neuronal inhibition in the hypothalamus [24]. Therefore, experimental approaches with exogenous leptin administration have been initiated in obese persons in order to limit food intake [25]. However, these approaches have failed due to impaired leptin function in obesity and its diverse regulation in various organs and tissues [26]. Obese individuals do not suffer from a lack of leptin; rather, they display higher circulating levels of leptin. In our study, including overweight or obese persons with at least one criterion of the metabolic syndrome, median baseline serum leptin concentrations of 18.0 µg/L [12.2; 25.5] were measured in females and 6.6 µg/L [4.0; 9.7] in males with tending higher levels in those with prediabetes. This is in line with earlier observations showing that in persons with the metabolic syndrome, 6-fold higher plasma leptin concentrations have been measured compared to controls [27]. However, in an obese or over-fed state, leptin lacks the ability to suppress food intake and to reduce body weight [25]. Thus, elevated leptin levels, defined as peripheral hyperleptinemia, are associated with leptin resistance and impaired leptin signalling in the brain [23]. Elevated plasma leptin levels have been shown to be the predominant cause for leptin resistance [6], resulting in chronic overstimulation of the leptin receptor and associated SOCS-3 induction [28]. Additionally, carbohydrate overfeeding [29], inflammation markers [30], impaired leptin transport across the blood–brain barrier, disturbed leptin signal transduction in neurons, and hypothalamic inflammation [31] are involved in this process.

High-protein, low-glycaemic meal replacement not only led to a significant weight loss but also to reduced leptin levels with lowest values after 1 month. This is in line with the previously reported U-shaped curve of carbohydrate consumption [17], estimating lowest carbohydrate ingestion during the first month with the most intensive meal replacement. Moreover, Zhao et al. [32] showed that leptin reduction during weight loss restored hypothalamic leptin sensitivity and led to reduced food intake, increased energy expenditure, and improved insulin sensitivity. Conflicting results have been reported concerning the question of whether higher plasma leptin levels at baseline would be predictive for weight regain [33] or not [2]. Our data confirmed that it was not the baseline leptin concentration, but an early and distinct leptin lowering [33,34] within the first month of intensive meal replacement that was predictive for long-term weight loss and this is in line with other dietary intervention studies demonstrating lower leptin levels in subjects without weight regain [35]. By using the meal replacement, not only effects on leptin and weight, but also an improvement in blood pressure could be observed [19], which could be explained by the fact that leptin mediates the relationship between fat mass and blood pressure [22]. In a previous work, we showed that the reduction of fasting insulin played an important role in weight reduction [18] and in combination with leptin we could demonstrate that those people with the strongest leptin reduction and tendencial higher insulin reduction had lost weight most effectively. This might be explained by the regulatory feedback loop between leptin and insulin signalling. In pancreatic β-cells leptin inhibits insulin biosynthesis and secretion [36], while insufficient leptin signalling in the hypothalamus induces peripheral hyperinsulinaemia. Insulin, in turn, stimulates leptin secretion from adipose tissue [37] and is supposed to regulate leptin secretion during weight loss [38].

The question remains why circulating leptin levels became reduced by a high-protein, low-glycaemic meal replacement. A limitation in this context is that only serum leptin concentrations had been measured. Genetic information might have offered further insight since it had been shown that polymorphisms of the leptin receptor gene are able to inhibit the decrease of serum leptin levels during dietary intervention [39] and are associated with blood lipids [40]. Therefore, we could just speculate about potential mechanisms, especially in other tissues. However, there are several hints that insulin-mediated inflammatory processes in the adipose tissue play an important role in leptin regulation. First, it is well known that obesity is associated with adipose tissue inflammation [41], activation of macrophages [42], and increased circulating plasma levels of pro-inflammatory cytokines [43]. Second, as shown before [44], baseline levels of serum leptin were not only positively correlated with body weight, BMI, and fat mass, but also with the inflammation markers IL-6 and CRP. During meal replacement, circulating levels of insulin and both inflammation markers became reduced [18]. Third, a positive correlation between leptin levels and the number of immune cells in the white adipose tissue had been documented [27] and immunostaining of white adipose tissue demonstrated half lower leptin signals in adipocytes but 5-fold higher leptin staining in adipose tissue mast cells from obese patients compared to lean patients [30]. Fourth, stimulation experiments with IL-6, tumour necrosis factor (TNF)-α, and insulin were able to provoke mast cells to secrete leptin [30]. Therefore, it might be concluded that in obese persons elevated levels of circulation leptin might not only result from increased secretion from adipocytes, but also from insulin-stimulated immune cells [30,38]. Fifth, the intervention group as well as data from published studies [21,45] showed a relative greater than expected reduction in serum leptin (−41% in females and −54% in males after 1 month) compared to fat mass loss (−7% and −9%), which supports the hypothesis that leptin secretion by immune cells within the adipose tissue might also be reduced. Finally, since insulin is supposed to drive subclinical inflammation, we propose that single components of the meal replacement, such as bioactive peptides with antioxidative, anti-inflammatory, and immunomodulatory properties [46], the carbohydrate reduction during intensive meal replacement itself [29], and the reduced carbohydrate-mediated insulin secretion might have contributed to diminished subclinical inflammation [18], and thus to lowered circulating leptin levels.

## 5. Conclusions

In the ACOORH trial lifestyle intervention, starting with a high-protein, low-glycaemic meal replacement was more effective in weight and leptin reduction than lifestyle intervention alone. Thereby, early and strong leptin and insulin reduction are predictive for long-term weight loss.

## Figures and Tables

**Table 1:** Baseline characteristics.

Parameters	Female		Male	
	Control Group (*n* = 80)	Intervention Group (*n* = 195)	Control Group (*n* = 54)	Intervention Group (*n* = 98)
Prediabetes (n)	30 (37.5%)	66 (33.8%)	10 (18.5%)	26 (26.5%)
Age (years)	52 ± 9	51 ± 10	50 ± 10	50 ± 8
Body mass index (kg/m^2^)	31.7 ± 2.4	31.5 ± 2.5	31.3 ± 2.4	32.1 ± 2.0
Weight (kg)	87.7 ± 9.1	86.5 ± 10.7	103.1 ± 9.1	104.1 ± 11.5
Fat mass (kg)	39.3 ± 6.0	38.2 ± 6.5	34.1 ± 6.2	34.8 ± 6.2
Lean body mass (kg)	48.4 ± 5.0	48.3 ± 5.7	68.9 ± 6.8	69.2 ± 7.4
Fasting insulin (µU/mL)	13.6 ± 6.9	14.6 ± 8.9	18.0 ± 10.7]	18.5 ± 11.0
Leptin (µg/L)	18.6 [13.7; 27.3]	17.6 [12.1; 24.9]	6.3 [3.3; 9.6]	6.8 [4.2; 9.8]
Leptin/fat mass (µg/L·kg)	0.5 [0.3; 0.7]	0.5 [0.3; 0.6]	0.2 [0.1; 0.3]	0.2 [0.1; 0.3]
Prediabetes (n)	30 (37.5%)	66 (33.8%)	10 (18.5%)	26 (26.5%)
Age (years)	52 ± 9	51 ± 10	50 ± 10	50 ± 8

Shown are mean ± standard deviation or median [interquartile range]. Chi-square and Mann Whitney tests were used for comparisons between groups.

**Table 2:** Associations between baseline leptin and baseline parameters.

	All (*n* = 427)		Female (*n* = 275)		Male (*n* = 152)	
Parameters	r	*p*	r	*p*	r	*p*
Sex	−0.65	**<0.001**	-	-	-	-
Prediabetes	0.15	**0.002**	0.11	0.064	0.08	0.338
Age	0.02	0.712	−0,02	0.792	0.03	0.703
Body mass index	0.24	**<0.001**	0.38	**<0.001**	0.29	**<0.001**
Body weight	−0.28	**<0.001**	0.24	**<0.001**	0.19	**0.017**
Fat mass	0.46	**<0.001**	0.38	**<0.001**	0.44	**<0.001**
Lean body mass	−0.54	**<0.001**	−0.01	0.899	−0.09	0.265
Fasting insulin	−0.02	0.685	0.18	**0.003**	0.20	**0.015**
Interleukin-6	0.07	0.225	0.07	0.319	−0.04	0.693
C-reactive protein	0.19	**<0.001**	0.17	**0.010**	−0.06	0.526

Bold *p*-values represent statistical significance. Spearman correlation was performed.

**Table 3:** Associations between leptin and weight or fat mass.

Parameters	Control Group (*n* = 134)				Intervention Group (*n* = 293)			
	r	*p*	ß	*p*	r	*p*	ß	*p*
**Leptin (baseline)**								
Body weight at baseline	−0.31	**<0.001**	−0.08	0.237	−0.18	**0.002**	−0.00	0.980
Δ weight after 1 month	0.09	0.312	0.00	0.883	0.12	**0.035**	0.01	0.571
Δ weight after 3 months	0.09	0.296	0.02	0.869	0.02	0.679	−0.03	0.677
Δ weight after 6 months	0.06	0.470	−0.04	0.724	−0.02	0.774	−0.07	0.351
Δ weight after 12 months	0.12	0.169	0.05	0.169	−0.04	0.500	−0.05	0.674
Fat mass at baseline	0.48	**<0.001**	0.12	0.271	0.41	**<0.001**	0.19	**0.007**
Δ fat mass after 1 month	0.15	0.075	0.04	0.605	0.07	0.243	0.04	0.445
Δ fat mass after 3 months	0.15	0.090	0.01	0.844	0.03	0.587	0.01	0.990
Δ fat mass after 6 months	0.11	0.209	−0.01	0.859	0.01	0.970	−0.03	0.572
Δ fat mass after 12 months	0.15	0.084	−0.03	0.657	−0.04	0.508	−0.06	0.244
**Δ Leptin (after 1 month)**								
Δ weight after 1 month	0.32	**<0.001**	0.34	**<0.001**	0.24	**<0.001**	0.33	**<0.001**
Δ weight after 3 months	0.38	**<0.001**	0.40	**<0.001**	0.35	**<0.001**	0.39	**<0.001**
Δ weight after 6 months	0.41	**<0.001**	0.44	**<0.001**	0.36	**<0.001**	0.39	**<0.001**
Δ weight after 12 months	0.31	**<0.001**	0.36	**<0.001**	0.32	**<0.001**	0.33	**<0.001**
Δ fat mass after 1 month	0.20	**0.018**	0.27	**0.002**	0.22	**<0.001**	0.25	**<0.001**
Δ fat mass after 3 months	0.27	**0.002**	0.34	**<0.001**	0.34	**<0.001**	0.37	**<0.001**
Δ fat mass after 6 months	0.36	**<0.001**	0.41	**<0.001**	0.33	**<0.001**	0.36	**<0.001**
Δ fat mass after 12 months	0.29	**0.001**	0.37	**<0.001**	0.29	**<0.001**	0.30	**<0.001**
Δ weight after 1 month	0.32	**<0.001**	0.34	**<0.001**	0.24	**<0.001**	0.33	**<0.001**
Δ weight after 3 months	0.38	**<0.001**	0.40	**<0.001**	0.35	**<0.001**	0.39	**<0.001**

Bold *p*-values represent statistical significance. Spearman correlations and multivariable linear regression analyses with adjustment for BMI, age, and sex were performed.

## Data Availability

The datasets generated during and/or analysed during the current study are not publicly available but are available from the corresponding author on reasonable request.
